# “Women and Opioid Addiction: Clinical Characteristics and Social Challenges”

**DOI:** 10.1192/j.eurpsy.2026.10809

**Published:** 2026-07-31

**Authors:** K. Taleb, L. Fatiha, B. Zineb, E. O. Fatima

**Affiliations:** 1 Aeazi Psychiatric Hospital; 2 Arazi Psychiatric Hospital, Salé, Morocco

## Abstract

**Introduction:**

Problematic opioid use is a major public health concern, with significant medical, psychiatric, and social consequences. In women, biological and psychosocial specificities influence addiction, comorbidities, and access to care.

**Objectives:**

To investigate the specific characteristics of problematic opioid use among women enrolled in a methadone program.

**Methods:**

A descriptive, cross-sectional, retrospective study of women followed in a methadone program. Data collected included age, duration of addiction, comorbid medical and psychiatric conditions, socio-professional status, type of opioid, and treatment history. Analysis was performed using Excel 2021.

**Results:**

Women had a younger median age and a rapid progression to dependence. Psychiatric comorbidities (depression, anxiety, trauma-related disorders) were frequent. Social and professional integration difficulties were pronounced, with substantial family responsibilities. Participation in the methadone program improved clinical and social stabilization but was influenced by socio-familial context.

**Conclusions:**

Problematic opioid use in women is characterized by increased vulnerability and specific needs. Integrated care combining substitution therapy, psychiatric follow-up, and social support is essential to optimize reintegration.

**Disclosure of Interest:**

None Declared

